# Shared community perspectives on how gender shapes coronary heart disease healthcare in Australia: a participatory systems thinking approach

**DOI:** 10.3389/fcvm.2026.1907168

**Published:** 2026-09-23

**Authors:** Tiana Felmingham, Helen Brown, Sarah Gauci, Kieva Richards, Dan Xu, Steven Allender, Sean Randall, Susan Brumby, Rachel Huxley, James J. Lucas

**Affiliations:** 1 School of Health and Social Development, Deakin University, Geelong, VIC, Australia; 2 Institute for Health Transformation, Deakin University, Burwood, VIC, Australia; 3 Global Centre for Preventive Health and Nutrition, Institute for Health Transformation, Deakin University, Geelong, VIC, Australia; 4 Institute for Physical Activity and Nutrition, Faculty of Health, Deakin University, Burwood, VIC, Australia; 5 School of Medicine, Faculty of Health, Deakin Food and Mood Centre, Deakin Institute for Mental and Physical Health and Clinical Translation (IMPACT), Deakin University, Geelong, VIC, Australia; 6 Department of Community and Clinical Health, School of Allied Health, Human Services, and Sport, La Trobe University, Bundoora, VIC, Australia; 7 Curtin School of Population Health, Faculty of Health Sciences, Curtin University, Bentley/Perth, WA, Australia; 8 Curtin Medical School, Faculty of Health Sciences, Curtin University, Bentley/Perth, WA, Australia; 9 The First Affiliated Hospital, Sun Yat-Sen University, Guangzhou, China; 10 National Centre for Farmer Health, Deakin University, Western District Health Service, Hamilton, VIC, Australia; 11 School of Medicine, Deakin University, Waurn Ponds, VIC, Australia; 12 Faculty of Health, Deakin University, Melbourne, VIC, Australia

**Keywords:** community, coronary heart disease, gender, service delivery, systems thinking

## Abstract

**Introduction:**

Coronary heart disease (CHD) remains one of the leading causes of death in Australia, yet women experience persistent inequities in diagnosis, treatment and recovery.

**Methods:**

A community-based systems thinking approach was applied using two, three hour online group model building workshops. Participants (*n* = 9) included patients, practitioners and advocates. A causal loop diagram was developed to map participant perspectives of gendered influences on CHD care. Analysis of the causal loop diagram was undertaken by two researchers who examined interconnections between factors and domains drawing on methods from system dynamics.

**Results:**

This study documents the complexity of embedded notions of gender and how these influence patient care in CHD in Australia. The findings demonstrate how professional knowledge, gender bias, community attitudes, and service delivery structures combine in unique ways to reinforce or disrupt inequities in CHD care, particularly for women, affecting the ability for the provision of individualised, tailored CHD care.

**Conclusions:**

Gendered assumptions during diagnosis, treatment and recovery influence CHD care, with specific impacts on women, resulting in misdiagnoses or delayed diagnosis.

## Background

Coronary Heart Disease (CHD) is the most common type of cardiovascular disease, highlighted by US data showing CHD accounting for 9,440,000 deaths in 2021 and for 185 million disability-adjusted life years lost (1). While historically, CHD has been viewed as a male disease, it is a leading cause of cardiovascular death in both men and women worldwide (2). Despite many campaigns to increase awareness of the impacts of CHD for women, disparities in prevention, diagnosis and treatment for women with CHD have yet to be sufficiently addressed.

A variety of factors are agreed to contribute to the disparity in CHD outcomes between men and women. Sex-based differences (e.g., such as anatomical and hormonal variations) influence disease presentation and progression, while gender-related factors (e.g., cultural norms and socioeconomic status) affect healthcare access and treatment (3). Women have been underrepresented or even excluded from clinical trials investigating CHD treatments (3), leading to gaps in understanding treatment safety and efficacy in women. Additionally, women are more likely than men to experience adverse outcomes due to barriers to accessing timely and appropriate care (4).

Individual access to health care services in Australia is influenced by a range of factors including geography, socioeconomic status, cultural background and health system organisation. These determinants extend to the diagnosis and treatment of CHD, contributing to inequities in who receives timely and effective care.

The patient journey for CHD has been evidenced to differ for men and women, contributing to disparities in outcomes. Evidence has shown gendered differences at the onset of symptom phase, including help-seeking behaviours, where men are likely to seek help earlier (2). Similarly, when patients reach medical care, women have reported perceived delays in diagnostic tests and treatment (2). Once in acute care, further disparities are evident. Women are less likely to undergo coronary angiography, catheterisation or revascularisation, are treated less promptly, and are less likely to receive optimal pharmacological therapies (4–6). Women also tend to have worse outcomes in hospital (4). These delays are thought to be driven in part by bias in both patients and healthcare professionals not considering CHD to be a disease that affects women.

This bias extends to the recovery period, with women being less likely to be referred to essential secondary prevention programs such as cardiac rehabilitation (7), and services shown to improve quality of life and reduce recurrent events. In the longer term, women are less likely to maintain adherence to secondary prevention strategies, including lifestyle modification and cardioprotective medications, further compounding disparities in outcomes (8).

While sex- and gender-based disparities in outcomes are well documented, far less is known about the lived experience of women navigating CHD care. Understanding these experiences requires attention not only to individual risk factors or isolated decisions, but to how structural, professional, and cultural influences combine in complex ways. Systems thinking provides an approach to capture and make sense of this complexity. By engaging patients, practitioners and communities, systems methods can reveal how multiple interacting factors produce reinforcing feedback loops that sustain inequities and highlight potential leverage points for change (9).

### Aim

The aim of this study was to apply a community-based systems thinking approach to explore shared perspectives on the role of gender in CHD health care experiences and service delivery in Australia. It was anticipated the application of such an approach would provide unique insights into the system-level factors shaping gender (in)equities in CHD healthcare.

## Methods

### Design

A community-based systems thinking approach (10) was utilised to explore individual and group perspectives on the role of gender in CHD healthcare service delivery in Australia (e.g., diagnosis and treatment after a cardiac event). This participatory approach creates a visual model of connections between influencing factors and identifies how these relationships uniquely combine to contribute to or alleviate a complex issue of community concern over time, from the perspective of community members. In this study we asked participants about the role of gender in CHD health care experiences and service delivery in Australia. It is important to note that gender was not considered itself a “concern” that needs “alleviating”, but rather we sought to understand what positively or negatively shapes the role of gender represented by a system of factors of cause and effect.

Group model building (GMB)— an established method within systems thinking— formed the primary method of exploration in this study. GMB provides detailed rigorous methods that guide workshop content and sequencing, incorporating the important role of community stakeholders into understanding nuanced complexities. GMB provides a unique opportunity to explore group agreements about a problem, while involving participants in visually depicting the influences and interconnections that underpin a problem. GMB has been used in numerous studies that aim to identify influences of a problem, and its use continues to grow (11–13). One of the main outputs of group model building is the development of a visual diagram – a causal loop diagram (CLD) (14). A CLD is a consensus-based visual representation of community members' mental models about the cause-effect relationships influencing a complex social issue. The development of a CLD is important in this instance as it has the ability to capture the ways participants describe factors influencing CHD care, and particularly, how factors combine in unique ways to hold some of the challenges patients experience in place.

Using GMB, a CLD is developed by the participant group in an iterative workshop process. Activities within workshops are guided by structured scripts to first elicit factors (also called graphs over time), and secondly, make connections between factors (known as connection circles). This is then followed by a map review activity where participants are asked to reflect on changes and amendments they would like to make to the diagram. More detail about each activity is reported later in this section.

### Participants

Potential participants were invited to be part of the study via an invitation that was shared through professional and organisational networks and social media platforms. Participants who expressed interest were included in the study and provided with a plain language statement and signed a consent form to participate. Participants were provided with a $50 pre-paid gift card in recognition for the time contributed to the study. Participants required access to a screen, camera and microphone to be able to participate. Fourteen people consented to take part in the study, nine (64%) of whom then participated in the GMB workshop process. Eight people attended the first workshop in November 2021 and eight people attended the second in December 2022. There was one participant who took part in the first workshop, but not the second. Similarly, there was one participant who took part in the second workshop, but not the first. The reasons for non-participation were not recorded. These participant numbers are considered sufficient for the purposes of GMB and applying a systems thinking approach. This a qualitative systems study with foundations in social construction and an aim of surfacing perspectives of workshop participants (10). Demographic details of participants are presented in the results section.

### Ethics

Ethical approval for the conduct of the study was granted by the University's Human Research Ethics Committee (HEAG-H 142_2022).

### The group model building workshops

Participants were invited to participate in two consecutive GMB workshops, with content and structure drawn from documented group model building scripts (15) (Supplementary file 1). Workshops were held online using the Zoom platform (16), and were recorded, including the generation of an audio transcript. Participants were able to join from a place convenient to them. Each workshop was approximately three hours in duration and held during business hours. Workshops were delivered by a facilitation team who are also researchers or authors (TF, SG, AB), with combined experiences across clinical, research and community settings. All facilitators were assigned specific roles for each workshop reflecting conventions used in group model building practice. The facilitation team for the first and second workshop comprised of: the facilitator (TF), a modeller (SG), a note taker (AB) and psychosocial support person (AB). The facilitation team agreed that a psychosocial support person was a crucial component of the team, to ensure a trauma-informed approach and to provide support to participants in the event discussions about their diagnosis or treatment triggered additional trauma. While this support was made available, it was not required by participants.

Design of both workshops was led by JJL, based on their previous systems thinking work (11, 13). Deakin University's online modelling software, Systems Thinking in Community Knowledge – Exchange (STICK-E) (see: https://sticke.deakin.edu.au/) (17) was used to create a causal loop diagram (CLD) of participant perspectives of factors and causal relationships between factors in real-time. System boundaries were limited to views from people from Australia with either lived experience of CHD or CHD practitioners.

All facilitators held dual roles in workshop facilitation and as researchers. This is an important note as the combination of these roles may influence which participant insights are included or excluded, and their interpretation. To ensure participant perspectives were preserved, wording used in the diagram reflected wording used by participants during the workshop. Facilitators continually checked wording and connections with participants as part of the iterative workshop process.

#### GMB workshop 1

The format of the first workshop reflected two main scripts: (1) graphs over time and (2) connection circles. The first script, graphs over time, aimed to identify multiple factors that influenced individuals CHD diagnosis and care, specifically highlighting gendered experiences. In line with system dynamics conventions, participants were asked to consider how these factors changed over time and draw a corresponding graph. The facilitator (TF) then asked participants to share their top three to five graphs and factors, one by one, with the modeller (SG) adding each factor to the beginning stages of the CLD. This prioritisation step was important in enabling participants to share different views in a safe environment.

The following script, connection circles, aimed to identify relationships between the factors identified in the graphs over time activity. The facilitator (TF) encouraged participants to describe their experiences, to help elicit the sequencing of connections and extend the causal relationships identified beyond a connection between two factors. As each connection was identified by a participant, the modeller (SG) added a connecting arrow between one factor to another in real time. These arrows capture the direction and polarity of the cause and effect between connections, for example, if an increase or decrease in one factor led to an increase or decrease in a subsequent factor, was considered as each connection was added. As the CLD was formed, additional factors were identified by participants and added to the diagram. At the conclusion of the first workshop, a partially completed CLD had been produced.

#### Preparation for GMB workshop 2

After GMB workshop 1, the facilitation team reviewed the partially completed CLD and corresponding notes that were taken on the day to validate the content of the diagram. Any factors that may have been identified through discussion on the day, as reflected in the notes, but were not yet reflected in the CLD, were added to the diagram in a different colour, to distinguish them from the original CLD developed live in the first workshop.

#### GMB workshop 2

The format of the second workshop built on content from the first workshop, and consisted of two main activities, reviewing and finalising the diagram and identifying areas to intervene. The facilitator (TF) led participants through a review of the diagram by first identifying and describing the colour additions as a result of the reflection and notes from GMB workshop 1. The facilitator then encouraged participants to suggest changes to the diagram, focusing on areas they felt were missing or needed to be changed. The facilitator prompted suggestions from each participant to ensure everyone had the opportunity to contribute. Any discrepancies were discussed and resolved by the group to ensure final group agreement. Additional factors and connections were made during this process. Once finalised, participants were asked to consider where on the diagram they thought action had already focused in the past and mark these parts with a tick (“✓”). Participants could mark as many ticks as they wanted. Then, participants were asked what they felt was most important in focusing future actions by placing a heart (“♥”) on the area. Participants were limited to three hearts for this part of the activity.

### Analytical approach

The primary data source was the final CLD created at the end GMB Workshop 2, supplemented with the notes taken within each GMB workshop for further clarification of the CLD content. The factors within the CLD were reflexively discussed amongst the author group and inductively thematically grouped into key ‘domains'. This involved two researchers (who also facilitated the workshops) reviewing the diagram together, which involved identifying themes based on a combination of the reflective notes, observations during each workshop and the wording used by participants in the CLD. Discrepancies about themes were resolved in discussion as part of the review. The participatory GMB workshop activities and iterative process of comparing CLD drafts and notes within and between each GMB workshop with participants and the author group ensured accuracy and completeness of the CLD content in representing consensus amongst participants' mental models, as per Hovmand's recommendations (10). A summary of the workshop and analysis process is described in Figure 1.

**Figure 1:**
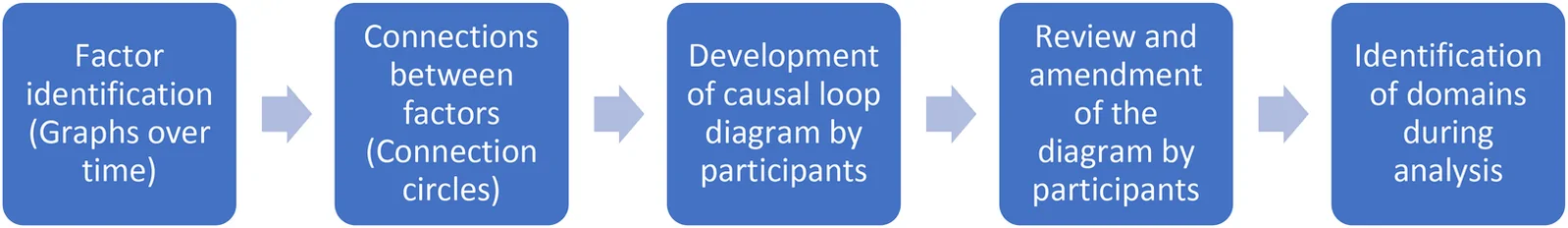
A visual workflow diagram illustrating the sequence of workshop processes, development of causal loop diagram and analysis.

## Results

### Participants' gender, role, and region

Of the nine participants, seven identified as women and two identified as men. Participants were located across Victoria, the Australian Capital Territory, New South Wales, and Western Australia. Seven participants disclosed their location; two were within a Modified Monash category 3 (MM3) area (‘Large Rural Towns'), one within an MM5 area (‘Small Rural Towns'), and four within an MM1 area (‘Metropolitan Areas') (18). The remaining participants did not disclose their location beyond the State level.

Three participants were individuals with lived experience of CHD and associated health care, four were CHD practitioners (e.g., doctor or allied health practitioner), one was a heart health advocate, and one was a practitioner-academic. It is important to note that some participants occupied more than one role, for example, being a practitioner and someone with CHD lived experience. In these instances, participants were asked to draw on multiple sources of lived experience. To maintain confidentiality in reporting however, the multiple positionalities by participant are not specified beyond this higher-level.

### Key domains (colours) and factors (rectangles)

After completion of the final CLD, the factors that participants identified through the GMB workshop process were thematically grouped by TF and SG under seven key domains, coloured and labelled: Knowledge of professionals (dark blue), funding (light green), gender bias (pink), service delivery – organisations (purple), individual (light blue), community (dark green), and professional practice (orange) (Figure 2). All of the factors appeared to be related to interpretation of gender roles, expression or identity.

**Figure 2:**
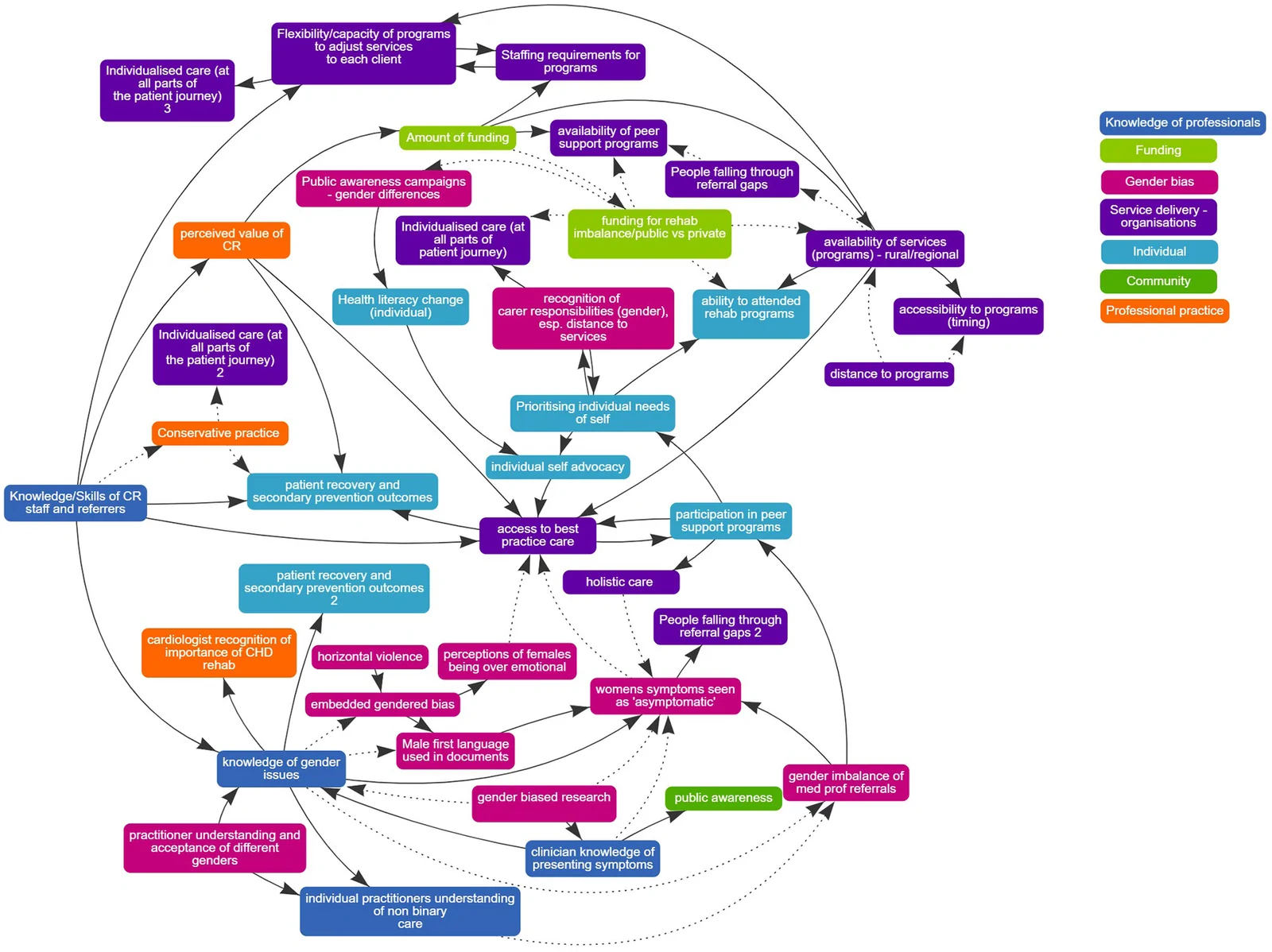
A visual workflow diagram illustrating the sequence of workshop processes, development of causal loop diagram and analysis. Causal Loop Diagram Outlining the Key Domains (colours) and their Associated Factors (rectangles) that Participants Identified in the GMB workshop process.Note. Domain legend in top right corner of figure: Knowledge of professionals (dark blue), funding (light green), gender bias (pink), service delivery – organisations (purple), individual (light blue), community (dark green), and professional practice (orange).

### Key cross-domain interconnections (the arrows connecting colours via rectangles)

Connections between key domains highlight the main stories described by participants. The following descriptions highlight important interconnections between multiple key domains. As demonstrated in the example below, a line between one rectangle, or factor, to another describes a connection between two factors highlighted by the group. An arrowhead helps describe where and how one factor influences another. The presence of a dotted arrow indicates how an increase in one factor causes a decrease in another factor (i.e., a negative polarity). This can also occur in the opposite direction, where a decrease in one factor leads to an increase in another. A solid arrow indicates how two factors influence each other in the same direction (i.e., a positive polarity), that is an increase in one causes an increase in another, or a decrease in one causes a decrease in another.

### Inter-domain connections

Over the course of the GMB workshop process, participants described the influence of cardiac rehabilitation staff and referrers' knowledge and skills (dark blue), which included their ‘knowledge of gender issues' (dark blue). Participants highlighted that as practitioners better understood concepts of gender and issues related to it, it helped to reduce ‘embedded gender bias' (pink) and subsequently, reduce the ‘perceptions of females being overly emotional' (pink), leading to ‘better access to care' (purple). Increasing the ‘knowledge of gender issues' (dark blue) was important to participants, with this also improving ‘practitioners understanding of non-binary care' (dark blue), which led to improvements in ‘gendered referrals from practitioners' (pink) (Figure 3).

**Figure 3:**
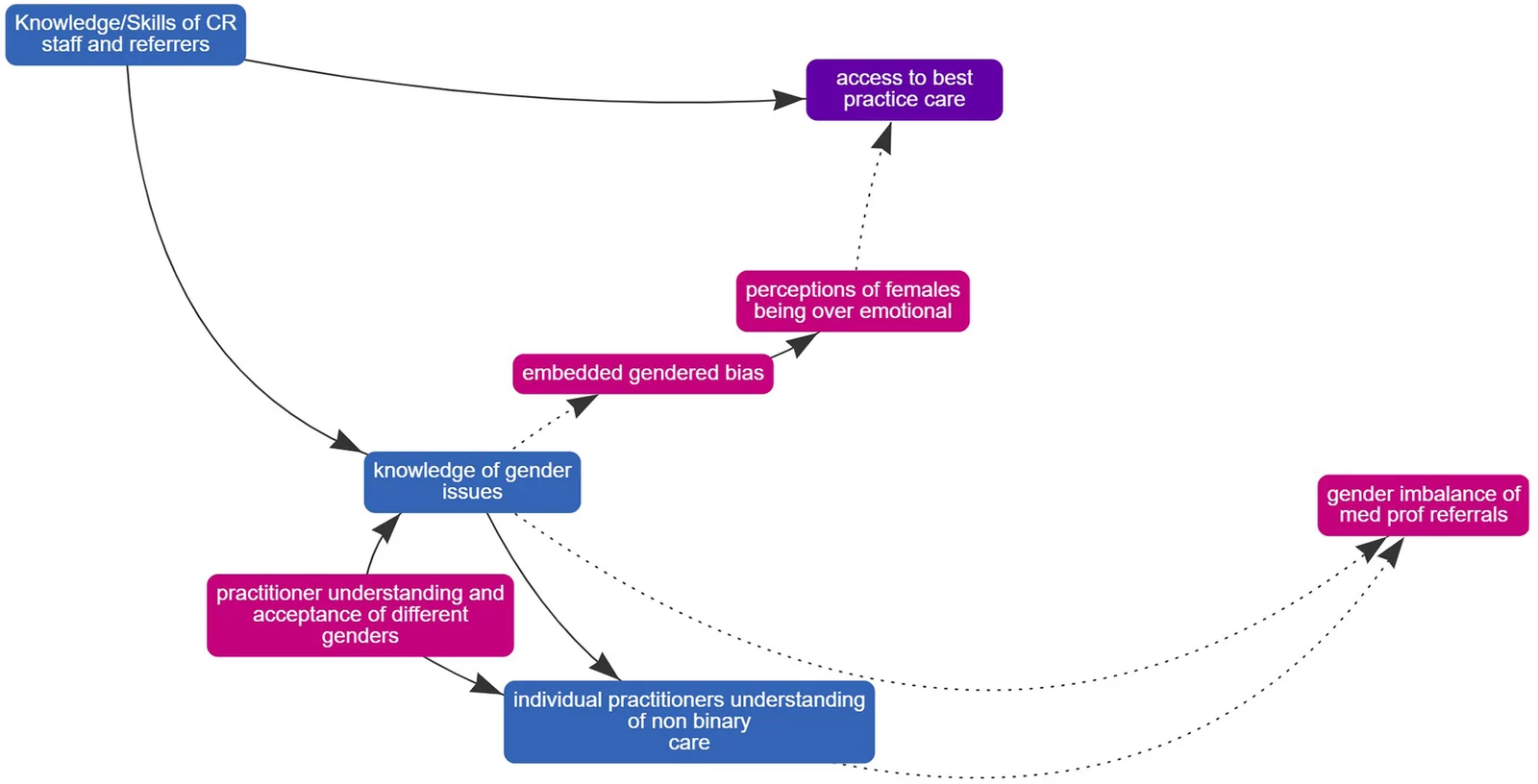
Connections between domains: knowledge of professionals (dark blue), gender bias (pink), and service delivery – organisations (purple).

Participants highlighted how the ‘gender imbalance of medical professionals referrals' (pink), ‘clinicians' knowledge of presenting symptoms' (dark blue), ‘gender biased research' (pink), and ‘male first language used in documents' (pink) all combined to influence the experiences women have of being dismissed when their 'symptoms are seen as asymptomatic' (pink) by professionals or women themselves. The participants described how these all stemmed from the knowledge and skills of cardiac rehabilitation staff and referrers in gender issues and in understanding different requirements of care (Figure 4).

**Figure 4:**
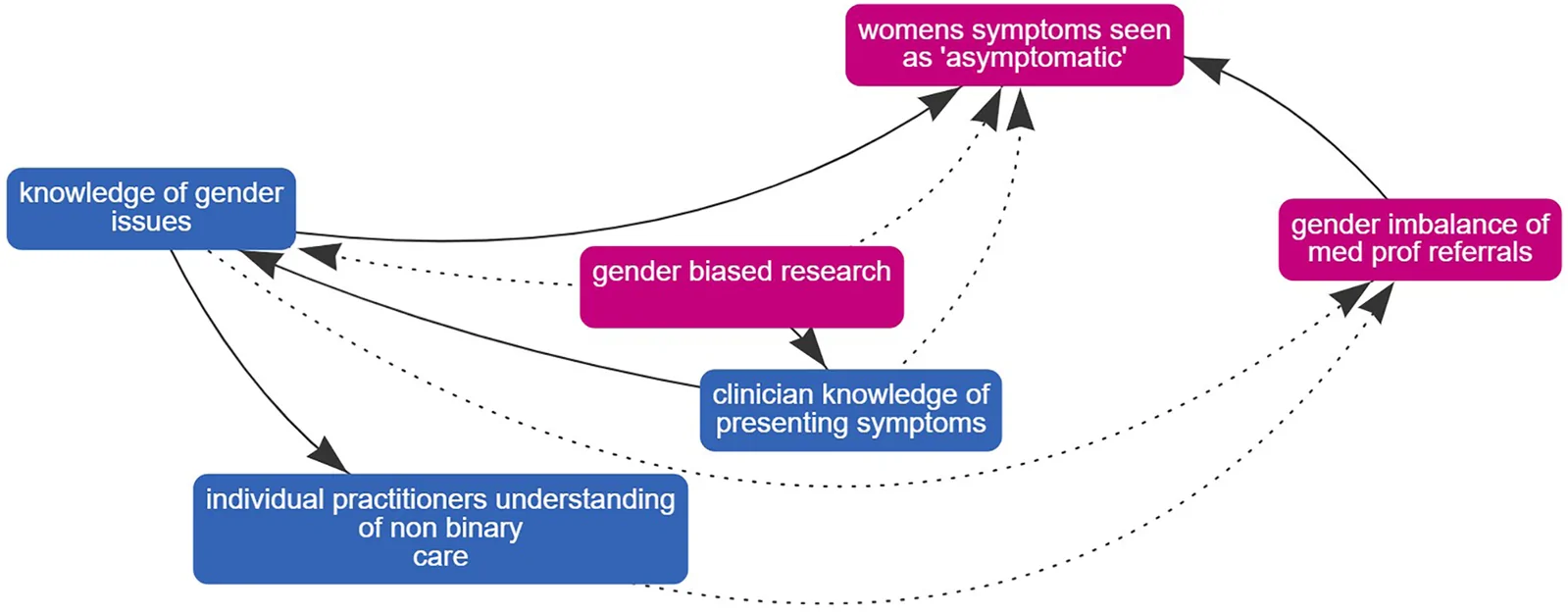
Connections between domains: knowledge of professionals (dark blue) and gender bias (pink).

While there was much focus in the discussion on practitioners' knowledge and how this translates into access to care, the group also highlighted the importance of practitioners' knowledge on the community's understanding and awareness of gender (green) when assessing presenting symptoms, through perceptions of authority and leadership of practitioners. The group also described how increases in the ‘knowledge and skills of cardiac rehab staff' (dark blue) was connected to professional practice (orange), specifically resulting in shifts away from ‘conservative practice' which encouraged approaches that embraced ‘individualised care' (purple) and, ultimately, increased ‘patient recovery and secondary prevention outcomes' (light blue). This discussion also highlighted the importance of practitioners' general knowledge and skills, and how this has an impact on the knowledge practitioners had of gendered symptoms and experiences. This in turn, influenced practitioners' ‘acknowledgement of the importance of CHD rehabilitation' (orange) for people of all genders, not only men (Figure 5).

**Figure 5:**
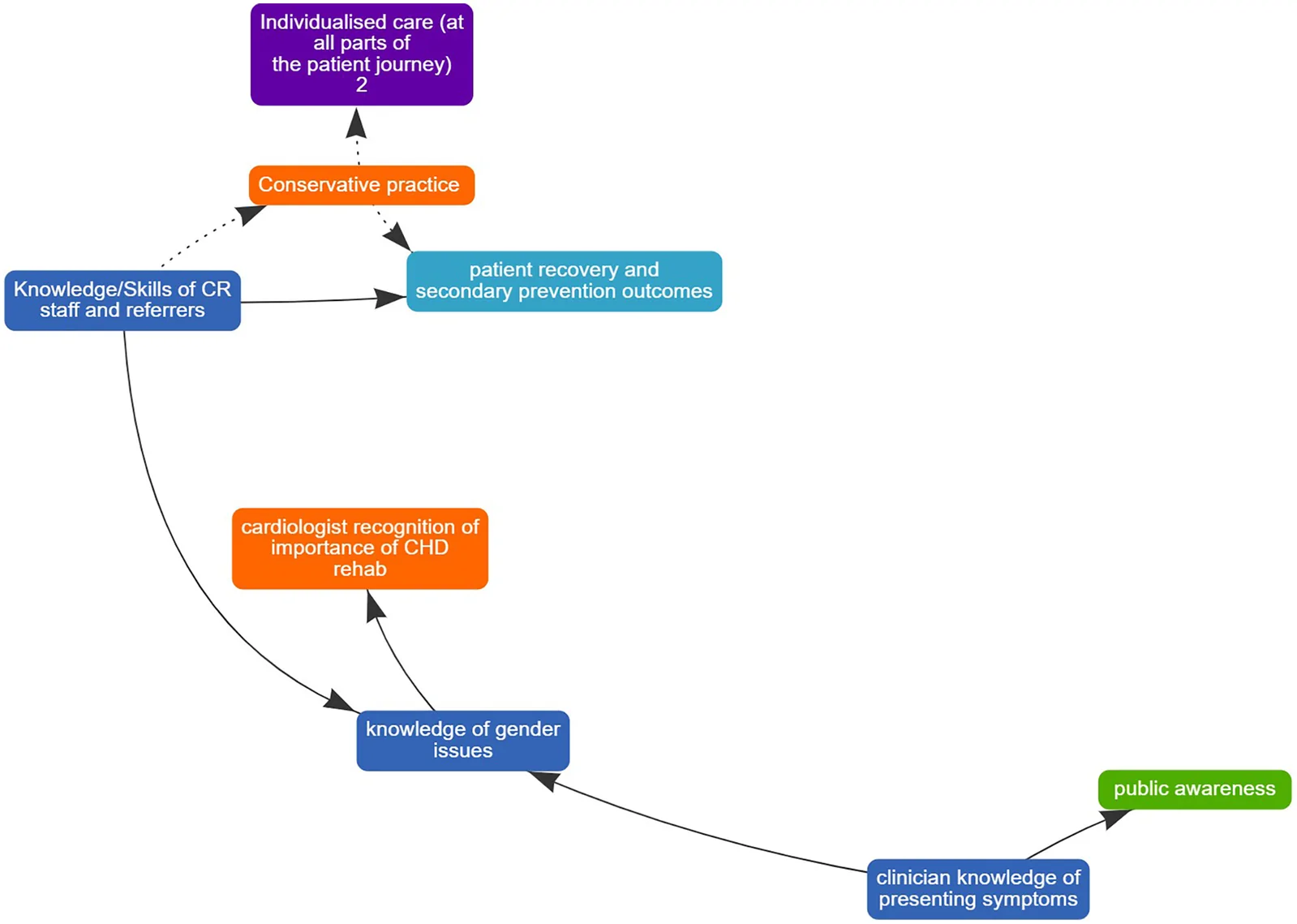
Connections between domains: knowledge of professionals (dark blue), community (dark green), professional practice (orange), service delivery – organisations (purple) and individual (light blue).

In addition, the participant group highlighted the importance of peer support programs in the first workshop, with one participant holding experience in the development and coordination peer support programs that helped CHD patients during recovery. Workshop participants described how participation in peer support programs' (light blue), was directly influenced by the referrals to attend the group. The group discussed how the limited number of women referred into these programs (pink), resulted in a limited number of women participating in peer support programs (light blue). The group noted how this had crucial implications for women's access to best practice care (purple). Participants noted the long-term impacts these combined relationships then had on access to best practice care over time and ultimately, patient recovery outcomes (light blue) (Figure 6). While concepts related to funding and unique influences of rural care were raised in the first workshop, these were not explored in depth by the group until the second workshop.

**Figure 6:**
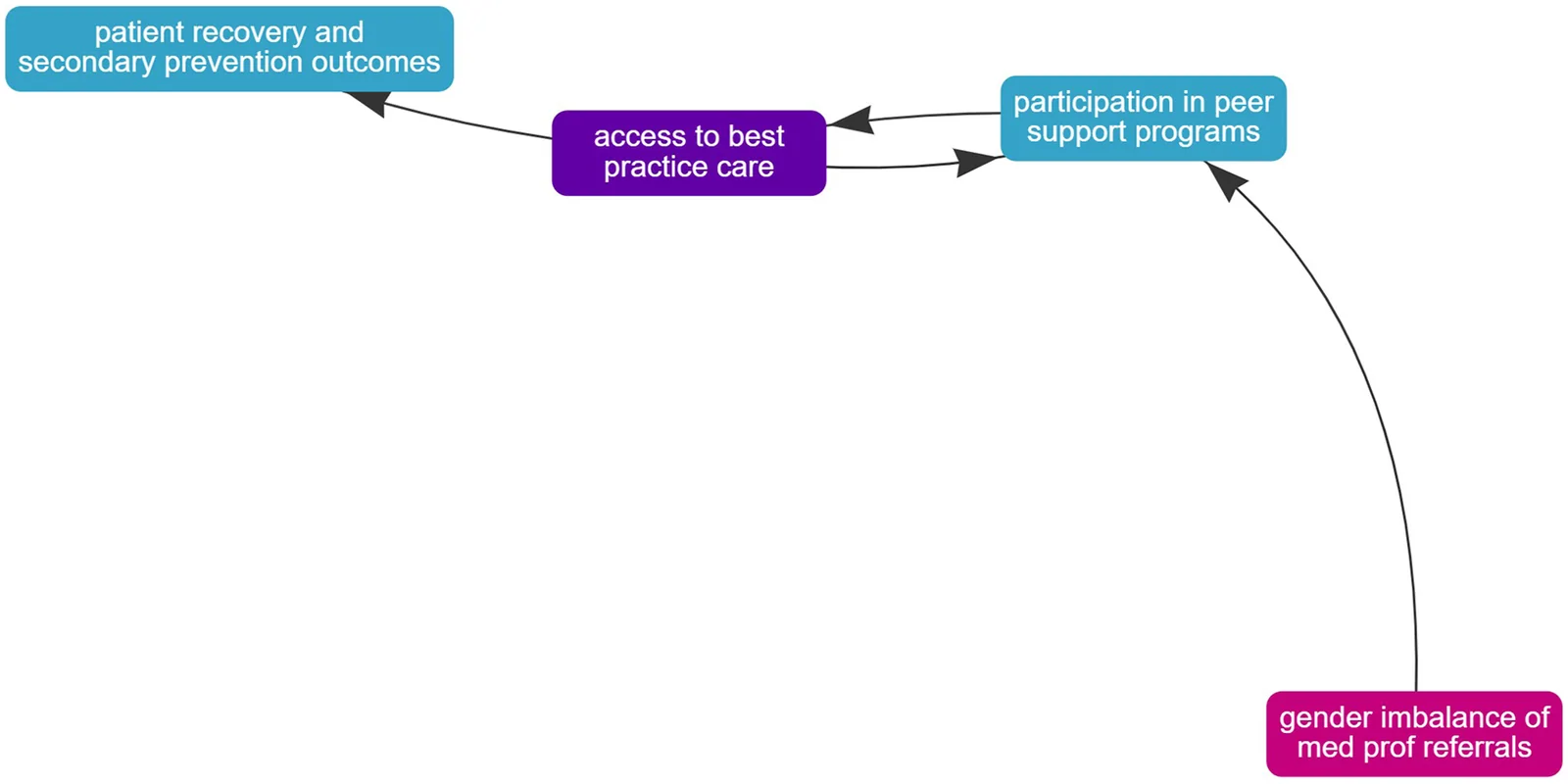
Connections between domains: individual (light blue), gender bias (pink) and service delivery – organisations (purple).

Participants also highlighted the importance of participation in peer support programs (light blue) in influencing individual patients' prioritisation of their own needs in addition to overall advocacy efforts (light blue). Participants discussed how the ability of individuals to advocate for their needs often meant they were more likely to attend rehabilitation programs, and in some cases, simultaneously having their increased carer responsibilities recognised, particularly where patients were women (pink) (Figure 7).

**Figure 7:**
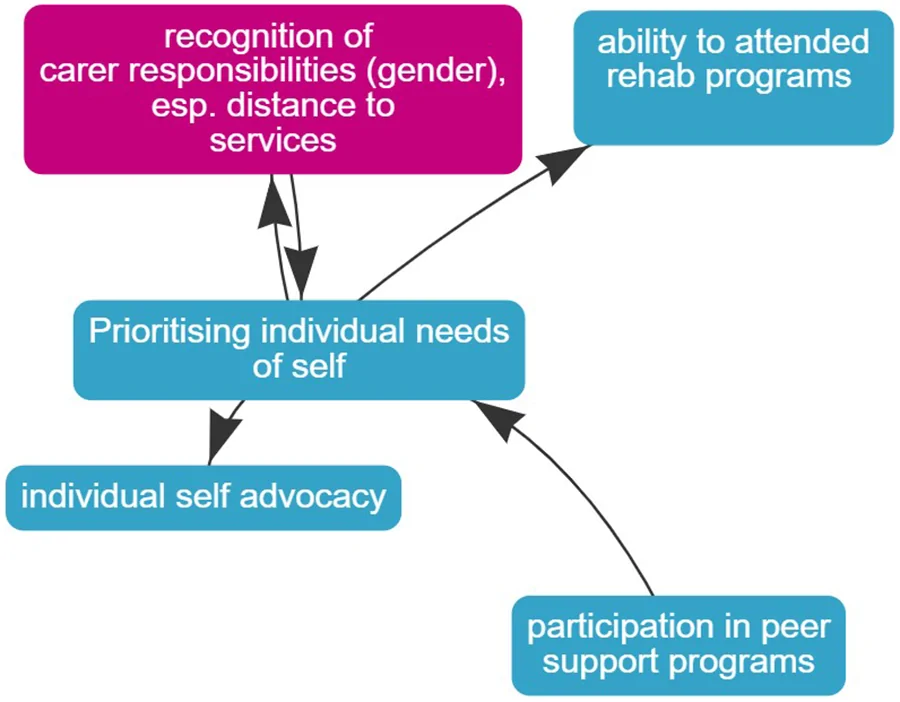
Connections between domains: individual (light blue) and gender bias (pink).

Lastly, understanding where people lived, particularly women, was highlighted as crucial for individualised care (purple), with participants describing the extra care arrangements (for example, organising care for others for over multiple nights) that are required for women with caring responsibilities. Recognition of carer responsibilities, distance and care logistics required to attend supportive post-event care, was described as directly impacting on an individual's ability to prioritise the care that they needed, ultimately affecting their access to best practice care (purple). Workshop participants described how funding allocations and access to flexible programming (light green) affected the care that people (particularly women) received. Participants highlighted how many people who lack access to flexible care locally (purple), and who also have caring responsibilities but need to travel to receive the care (pink), are ultimately forced to choose between the two. Caring responsibilities often take priority, meaning that women (mostly) are unable to prioritise or receive the care they need (light blue), even when they wish to follow through on clinicians' recommendations (Figure 8).

**Figure 8:**
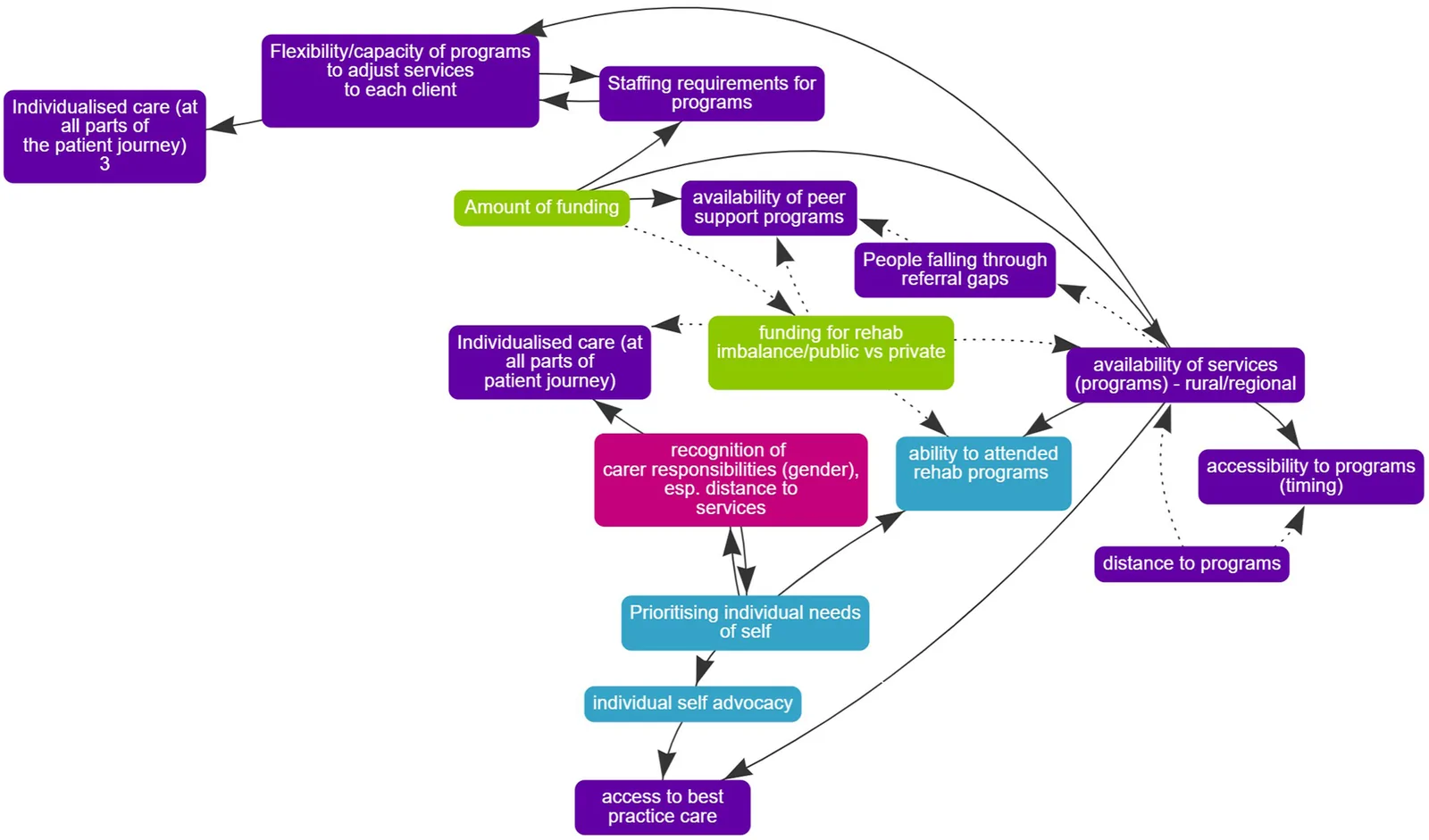
Connections between domains: individual (light blue), gender bias (pink) and service delivery – organisations (purple).

## Discussion

This study documents the complexity of embedded notions of gender and how these influence patient care with CHD in Australia, by documenting mental models of community members. Gendered factors and interrelationships affecting CHD care were identified across seven domains: professional knowledge, gender bias, funding, service delivery, individual, community, and professional practice.

A key finding in this study was how participants described where practitioners' knowledge of gender issues combined with their expressions of presenting symptoms which in turn shaped patients' access and experience of quality CHD care. In particular, this study highlighted how gendered assumptions that underlie clinical language, diagnostic frameworks, and research influence women's CHD experiences, where concerns are often not taken seriously affecting future referrals and provision of relevant information, resulting in greater misdiagnosis or delayed diagnosis.

Our findings highlight how participants described the intersection of geography with gender compound inequities in CHD health care. For women residing outside of major metropolitan centres, limited access to specialist cardiac care together with entrenched professional conservatism created experiences where women's symptoms were minimised or attributed to non-cardiac causes (e.g., stress). Participants shared that geography also influenced their access to CHD care. These findings support existing evidence that heart health outcomes of women are spatially mediated (19, 20), thus amplifying gender-based disadvantage within Australia's health system.

The participant-created CLD highlighted interconnected challenges for women in accessing peer support after a CHD event. Inclusive peer support programs could enhance visibility of women's health needs and lived experiences, but barriers to women's participation in such programs limits the visibility (and advocacy for) their needs and experiences. This finding is consistent with existing research (2, 21, 22), but where the study goes further is in highlighting these patterns as part of a broader, interconnected system rather than of individual—or program—level oversights.

These findings are consistent with existing evidence that women often experience dismissal by practitioners, delayed diagnosis, and lower cardiac rehabilitation referral rates compared with men (2, 8). A study by McSweeney (23) published two decades ago captured the diagnostic experiences of 40 women who had CHD symptoms, identifying five themes, some of which align with the themes identified in our study: awareness, seeking treatment, frustration, treatment decision and anger. The authors found that women's concerns were often not taken seriously with women reporting that they felt their CHD symptoms were ‘in their heads'. This aligns with the findings in our study that found women reported feeling dismissed with their symptoms being seen as asymptomatic. Our study extends on the findings by McSweeney in demonstrating the interconnected complexities that combine to hold these perceptions and assumptions in place, reinforcing gendered experiences of CHD care. While McSweeney's study was conducted almost two decades ago, our study shows there is still a need for a focus on disrupting the gendered assumptions that are embedded in CHD care if it is to improve.

The use of systems thinking to explore complexity of influences in clinical care has been documented in other studies (24–26). For example, Gillani et al. (25) recently documented barriers to physical, mental and dental healthcare in sexual and minority groups in the United States, also through the use of group model building and the development of a causal loop diagram. Their study found that individual experiences of discrimination and assumptions about individual needs influenced access to, and engagement in ongoing care, which is reiterated in the findings in our study. Our research differs from Gilliani by exploring assumptions about gender and extends on this by focussing on CHD specifically.

This study extends existing research to show that such gendered CHD experiences and outcomes are not simply the result of individual practitioner behaviour, but are embedded across interconnected professional, cultural, and organisational structures – further evidence of the need for system-wide approaches that recognise the role of gender in equity of CHD healthcare.

This is the first study to the author groups' knowledge that utilises a community-based systems thinking approach to understand the role of gender in CHD health care experiences and service delivery in Australia. The use of participatory research to understand complexity is a major strength of GMB workshops and provides a unique understanding of a complex public health issue.

This study includes a range of professional and lived experience perspectives, including those who hold both of these perspectives simultaneously. It also includes contributions from individuals across metropolitan and rural areas than spanned multiple Australian states. This diverse representation strengthens insights documented in the causal loop diagram.

This study was conducted in Australia, and while the findings provide insights into gendered experiences of CHD care applicable in other settings, the transferability of findings are limited to those locations with similar characteristics, where the provision of healthcare is generally stretched and underfunded but publicly available, operating in a relatively stable political and economic environment. This study documented perspectives of nine workshop participants. While diverse, this number may also limit transferability of results.

GMB requires time commitments from participants and is typically conducted with smaller groups. The resulting CLD is a function of who is selected or self-selects to participate. The GMB literature highlights how the content and insights presented in a CLD are shaped by those who are ‘in the room' (10). While this may be considered a limitation in terms of surfacing a small number of perspectives, the group focused facilitation techniques used in GMB ensures a there is a focus on managing power dynamics and creating group agreement and consensus, strengthening documented insights.

It is acknowledged that recruitment through professional networks and voluntary participation may have attracted individuals with greater awareness of gender-related issues in CHD care. However, the purpose of GMB is not to generate statistically representative estimates of prevalence, but rather to explore and visualise stakeholder mental models regarding a complex issue. Consistent with community-based systems thinking methods, the value of the approach lies in documenting how participants understand system structure and interactions, rather than establishing the frequency with which particular perspectives are held.

While facilitator-researcher dual roles may introduce the potential for influence during data generation and analysis, this is a recognised feature of participatory systems methods. Several safeguards were implemented including use of participant-generated language within the CLD, iterative participant review of factors and connections, and consensus-based validation of diagram content across workshops. Accordingly, the CLD remained primarily participant-generated rather than researcher-derived.

The possibility that dominant voices may influence group discussions is acknoweldged. However, structured GMB scripts are specifically designed to reduce this risk. Individual idea generation activities preceded group discussion, contributions were systematically elicited from each participant, and diagram amendments required group agreement. These processes were intended to support equitable participation and the incorporation of multiple perspectives.

The need for individualised, person-centred CHD care was important to participants in our study. Studies by Al-Smadi et al. (27), and Bairey et al., 2017 (28) highlight that social factors, including stigma or fear of judgment about weight, lifestyle or body size, contribute to delays in seeking care for women. Compounding this, systemic and conventional health care models overlook psychosocial determinants related to gender roles, expression and identity such as caregiving roles, chronic stress, mental health and discrimination (29, 30). Together, these factors underscore the need for an inclusive, person-centred approach in clinical and community care to reduce disparities and prevent misdiagnosis or delayed care in women's heart health. Person-centred care focuses on building a partnership or a collaboration with the client to engage in shared decision making. This approach values the clients, their perspectives, their knowledge, their abilities and experiences, allowing the provision of high quality, ethical, effective care (31).

## Conclusion and future perspectives

While our study explored experiences and perspectives at a national level, it will be important to understand gendered nuances between locations, as surfaced in our study. For example, gendered influences in metropolitan areas may differ from those in regional, rural or remote areas of Australia. Future research that focuses on differences across geographic locations may be more likely to generate action from local organisations than an approach from a national perspective. Future research could also consider how gender could be incorporated into strategies that promote individualised care.

Understanding how gender influences CHD care is crucial in the equitable management of CHD. The application of systems thinking ensures the complex nature of these influences are considered in the design of future diagnosis, treatment and recovery.

## Data Availability

The raw data supporting the conclusions of this article will be made available by the authors, without undue reservation.
