# Supplementary material for: Shared community perspectives on how gender shapes coronary heart disease healthcare in Australia: a participatory systems thinking approach
Source: Front Cardiovasc Med. 2026 Sep 23;13:1907168. doi: 10.3389/fcvm.2026.1907168 (PMC13642013; doi:10.3389/fcvm.2026.1907168)
Supplement: Supplementary file 1 [file Table1.docx]

**Session Agenda: First Workshop (180 minutes)**

| Stopwatch | Duration | Activity | Description |
| --- | --- | --- | --- |
| -0:30 | 30 mins | Room Setup | Members of the **Facilitation Team** arrange the room |
| 0:00 | 15 mins | Welcome &  Introduction to the workshop | The **Opener/Closer** welcomes participants and opens the meeting.  **Opener/Closer** introduces participants and the **Facilitation Team**, and gives a brief overview of the project and activities to be completed. |
| 0:15 | 60 mins | Graphs over Time | The **Facilitator** introduces the “Graphs over Time” exercise and shows the group how to complete a Graph over Time template using an example drawn on a whiteboard.  The **Facilitator** gives participants 10 minutes to fill in as many graph over time templates as they can to show  *what influences* ***gendered experiences*** *of* ***CHD service delivery (practitioners & organisations)*** *in our community?*  The **Facilitator** then instructs participants to work in small groups of two or three for a further 10 minutes to combine their graphs into one pile, prioritized from most important (at the top) to less important (lower down).  The **Facilitator** asks each group in turn to share the top graph from their pile, including the name and the trend over time. As each graph is shared, the **Modeller** enters the factor name into STICKE in preparation for the next script.  **Note Takers** are typing notes of the graphs as they are shared. |
| 1.15 | 60 mins | Connection Circles | The **Facilitator** introduces the connection circle activity and invites someone to identify a pair of factors around the circle that are connected in some way.  The **Modeller** enters the connection in STICKE (live on a projector, visible to participants) as the **Facilitator** is guiding the discussion. The **Note Takers** are typing notes of the stories as they are shared. |

| Stopwatch | Duration | Activity | Description |
| --- | --- | --- | --- |
|  |  |  | After 20 minutes, or once all factors around the circle have at least one connection, the **Facilitator** announces that we will switch to diagram view.  The **Modeller** switches to diagram view, and the process of identifying connections continues until the conclusion of the activity. A 5-minute break may be given to participants if the **Facilitation Team** would like to go “offline” to untangle the connection circle.  At the end of the activity, the **Facilitator** hands over to the **Opener/Closer** to end the session. |
| 2.15 | 15 mins | Closing | The **Opener/Closer** explains the next steps including a brief overview of the activities coming in the next session.  The **Opener/Closer** thanks participants for their time, invites them to stay after if they have more questions, and reminds them of the next meeting time/date. |
| 2.30 | n/a | Session Closed |  |
| n/a | 20 mins | Debrief | The **Facilitation Team** debrief the workshop, discussing what went well, what was challenging, and lessons for next time. |

**SCRIPT: Introduction to the workshop Session**

| **Duration** | 5-10 minutes |
| --- | --- |
| **Materials** | - Session agenda - Slides (optional) |
| **Roles** | **Opener/Closer** who can introduce the activity and its purpose, and is confident speaking to the participant group |
| **Steps** | 1. The **Opener** announces the start of the session.    - Acknowledgment of Country (or Welcome to Country if applicable and appropriate)    - Welcome the participants and thank them for attending.    - Briefly explains the background of the project, and the purpose for bringing community leaders/members into the project.    - If the session is taking place in unfamiliar room, inform participants of the location of restrooms, exits, etc. 2. The **Opener** begins the introductions:    - Introduce yourself, and then say that there are more members of the facilitation team in the room, and before we get to the participants, we want to let you know who we are.    - Each team member introduces themselves and describes their role.    - The opener then asks participants to introduce themselves, using an icebreaker activity if desired.    - The opener then describes the plan for the workshop session, when breaks will be, and asks if all participants are ready to begin. |
| **Original Script** | Originally documented by Timothy Hower [(thower@wustl.edu](mailto:(thower@wustl.edu) ), Krista Rux (krux@wustl.edu) and Peter Hovmand [(phovmand@wustl.edu](mailto:(phovmand@wustl.edu) ) for the Federal Reserve Bank Project, September 21, 2011 |

**SCRIPT: Graphs over Time**

| **Duration** | 30 minutes |
| --- | --- |
| **Materials** | - Stacks of A5 Graph over Time templates (5-10 per participant) - Textas - Computer & projector with STICKE set up |
| **Roles** | - **Facilitator** to work with the group and guide discussion - **Note takers** to capture notes on the factors as they are shared by participants - **Modeller** to enter factors into STICKE in preparation for Connection Circles script |
| **Steps** | 1. The **Facilitator** introduces graphs over time as a way to visually describe a problem, beginning with the reference mode (the complex problem of interest), describing how it has changed from the past until now – and the hoped and feared behaviours into the future. 2. The **Facilitator** prompts participants to consider *what influences* ***gendered experiences*** *of* ***CHD service delivery (practitioners & organisations)*** *in our community?* The **Facilitator** asks participants for examples of factors and picks one of the examples. A template for graphs over time is already drawn on the whiteboard, and participants have templates in front of them. The **Facilitator** explains that the factor they have chosen can increase, decrease, stay the same, or go up and down over time, and draws examples of each of these behaviours. The **Facilitator** asks the group which of these four behaviours best fits the example factor. When the group picks, the **Facilitator** erases the other three lines. The **Facilitator** then asks the group what they hope and fear the factor will do in the future, and draws lines accordingly. As the **Facilitator** does this example at the front of the room, each person can have a template in front of them and do their own along with the facilitator (using their own factor of choice). 3. The **Facilitator** then asks participants to draw one graph over time per piece of paper, for each thing they can think of that affects the problem. Participants are given 5-10 minutes to work individually. 4. After 10 minutes, bring the group back together.   The **Facilitator** instructs participants to work in groups of 2-3 to share their graphs with each other and choose the ones they think are most important. Participants are given 10 minutes to complete this task.   5. The **Facilitator** leads a round of sharing in the final 10 minutes of the activity, and groups take turns sharing their top factors. 6. During sharing the **Modeller** is adding the variables suggested into STICKE in preparation for connection circles. |
| **Original Script** | See “Graphs Over Time” script: https://en.wikibooks.org/wiki/Scriptapedia/Graphs_over_Time |

**SCRIPT: Connection Circles**

| **Duration** | 60 minutes |
| --- | --- |
| **Materials** | - Projector and screen - Computer running STICKE |
| **Roles** | - **Modeller** with some experience in STICKE - **Facilitator** with experience facilitating groups and some experience with building systems maps in STICKE - **Note Takers** to capture notes on the discussion during the activity |
| **Steps** | 1. The **Facilitator** introduces the connection circle script.   *“The goal of a connection circle exercise is to find the connections between different concepts or variables that contribute to or are affected by some issue—in this case what influences* ***gendered experiences*** *of* ***CHD service delivery (practitioners & organisations)*** *in your community.*  *We can start by taking two of the concepts you created in the first exercise. Can someone identify two factors that share a relationship?”*   2. The **Facilitator** clarifies from the participants the nature of the relationship between the factors (solid arrow, change in the same direction OR dashed arrow, change in opposite directions), and then the **Modeller** draws the appropriate connection in STICKE. The **Modeller** draws the links as the **Facilitator** is speaking, and STICKE is projected. 3. The **Facilitator** continues to ask for connections, focussing on extending the story captured in the first connection.   *“We have just made a connection between A and B – what else do we know about this story – is there anything that contributes to A, or can we think of anything that changes because of B?”*  Once participants have run out of connections for the initial story, or if a particularly meaningful story moves the discussion onto another pair of factors, return to the prompt above for the new story.  The **Note Taker** is recording the stories shared and conversations between participants.   4. Once most of the factors around the connection circle have at least one connection (if there are 1-2 factors that still need to be connected, draw participants’ attention to that factor and ask them to make the connection), the **Facilitator** says:   *“We will now switch to a view called diagram view, which will make it easier to read and understand this diagram.”* |

|  | 5. The **Modeller** then switches to diagram view, and the picture is rearranged. If the picture is very tangled and requires more effort to clarify – the **Facilitator** may suggest a 5-10 minute break. The **Modeller** proceeds to clarify the diagram. 6. The **Facilitator** asks for additional connections people can see in this view. In this view in particular, the **Facilitator** emphasizes feedback loops, encouraging participants to extend on existing stories, and close feedback loops if possible, rather than add connections more generally. |
| --- | --- |
| **Original Script** | See “Creating Causal Loop Diagram from Connection Circles” script: https://en.wikibooks.org/wiki/Scriptapedia/Creating_Causal_Loop_Diagram_ from_Connection_Circles |
